# The Informatics for Making Sense of the Genome: A Progress Report From the BioPathways Consortium

**DOI:** 10.1002/cfg.145

**Published:** 2002-04

**Authors:** Eric K. Neumann, Vincent Schachter

**Affiliations:** 1 Beyond Genomics 40 Bear Hill Road Waltham MA 02451 USA; 2 Hybrigenics 3-5 Impasse Reille Paris 75014 France


**Comparative and Functional Genomics**
*Comp Funct Genom* 2002; **3**: 115–118.
Published online 12 March 2002 in Wiley InterScience (www.interscience.wiley.com). **DOI:** 10.1002/cfg.145

**Review**

### The informatics for making sense of the genome: a progress report from the BioPathways Consortium

Eric K. Neumann[1] and Vincent Schachter[2]*
[1] *Beyond Genomics, 40 Bear Hill Road, Waltham, MA 02451, USA*
[2] *Hybrigenics, 3-5 Impasse Reille, 75014 Paris, France*

**Correspondence to:
Hybrigenics, 3-5 Impasse Reille,
75014 Paris, France.
E-mail: vschachter@hybrigenics.fr*

**Keywords: Biopathways; systems biology; metabolic pathways; regulatory networks; signal transduction; ontologies**

Received: 29 January 2002
Accepted: 29 January 2002

The sequenced human genome potentially offers a wealth of useful knowledge to researchers. The better part of this promise, however, is conditioned by the availability of this information in a form accessible to computational methodologies. Representations such as IUPAC[1], and formats such as FASTA have made it possible to develop and apply bioinformatics tools to genomic data. Collections of analytical tools developed so far assume that information is represented in a standard way, thereby greatly increasing the reusability of code, and reducing the time necessary for the development of new methods. While genomics research has had much to gain from this common structure, the fact that some essential standards are still lacking is an impediment to the commercialization of new bioinformatics tools.

A reiteration of this effort is much needed for the post-genomics age, but the task required goes far beyond analyzing sequences and molecular structures. The challenge now facing researchers is to represent, analyze, and model molecular interactions and biochemical systems, in an integrative approach commonly known as systems biology [3], in order to determine how groups of proteins function within the context of a cell. These are very new areas for life sciences informatics, demanding novel representations and corresponding algorithms. Similar – albeit arguably simpler – modeling problems have been encountered in other phenomenological fields, however, such as engineering or telecommunications, and theoretical tools have been developed to tackle them. Some of these tools can be adapted to the context of systems biology, and can help in turn organize the scientific effort toward a coherent understanding of biological mechanisms. The BioPathways Consortium [1] has a dual mission: to propose and advance the post-genomic informatics to this next level of scientific research, and to help the industry adopt practical guidelines and standards. We present below some of BPC's activities since its formation in June 2000, and offer a vision of how our efforts will support systems biology research.

Data formats for DNA and protein sequences have been developed over the last 30 years, and are usually based on binary or string representations, such as IUPAC. While sequence representations have been standardized, other associated components of information, such as functional descriptions, are not. Recently, the GeneOntology consortium (**http://www.geneontology.org**) has organized a system of

[1]International Union of Pure and Applied Chemistry

Copyright © 2002 John Wiley & Sons, Ltd.

functional, compartmental, and process descriptors into a taxonomy. This effort has resulted in a controlled vocabulary, common to several genomes, that can be incorporated into annotations. Yet a more complete language of descriptors is still needed to describe how proteins and genes interact and associate with each other.

Life sciences research has now entered the 'post-genomics age', where the focus is moving from isolated molecular structures to systems of interacting molecules. Representations for transcriptomic and proteomic data are actively becoming standardized (MAGE-OM, I3C) to handle the onslaught of data from newly emerging high-throughput technologies, such as chip arrays and mass spectrometers. Yet the capture of a formal interpretation of this data also requires a descriptive language.

Scientists are now faced with the challenge of having to denote biochemical processes and dynamic behavior using formal models. These models are often based on graphs, mathematical entities composed of sets of vertices connected by edges [2]. Graphs may be used to represent causal flow (directed) in biochemical processes, or to describe non-directional molecular interactions and associations (undirected) between two or more molecules. Graph theory is a fairly mature sub-field of discrete mathematics and theoretical computer science: graphs, as mathematical objects, are well understood. For instance, it is well known that certain kinds of graph problems are computationally intractable. To illustrate how fast graphs can grow in complexity, consider a 4-node labeled graph, which has $2^{4\times 4} \Rightarrow 65536$ possible directed edge combinations, while a 10-node graph has $1.27 \times 10^{30}$ combinations. Clearly a simple bio-molecular system represented as a graph could overwhelm even the most powerful supercomputers today. If one assumes the human genome encodes 100 000 proteins, the total number of 2-way interactions possible between all proteins is about $10^{30,103}$! In other words, how we represent biological systems and the computational techniques we use to analyze them will have a strong bearing on how well we can elucidate and model such systems.

The term ***pathway*** is often used to describe biochemical systems. It is, however, fraught with multiple connotations that many researchers believe are misleading. Many assume that it interjects a linear model of biochemical events, while others interpret it as more general, including such forms as directed acyclic graphs [2]. Several researchers have suggested using the alternative terms 'network' or 'circuitry'. Yet it seems that many have grown accustomed to the term pathways to mean a consequential system of interactions, even though feedback loops may be part of such a system. The BioPathways Consortium has advocated the use of this term in its name for this reason, but stresses that it has a much larger interpretation to be as inclusive as possible.

A characterization for some of the categories of pathway information is now in order. *metabolic pathways*, such as those found on the classical Boehringer-Mannheim chart, illustrate how substrates are catalytically altered by enzymes to produce products along a path of biochemical reactions [4]. *Signal transduction pathways* involve cascades of molecular interactions and activations, which are used to transmit and amplify signals relevant to the cell [8]. *Gene regulatory networks*, forming the last category, represent the circuitry by which gene expression activation spreads to downstream genes, affecting their expression. In actuality, a gene is regulated by the interaction between active transcriptional factors in the nucleus and the sequences surrounding a gene known as *cis*-elements. These models describe logic circuits on how genes control each other, and subsequently the cellular states [3]. They can be construed as abstract views of networks of actual physical interactions.

A related category is that of protein interaction maps, describing networks of physical interactions between proteins or protein domains. One way to interpret these maps is to see them as partial and incomplete descriptions of pathways: although the exact nature, causal flow, separation into independent pathways or temporal sequence of interactions are unknown, the template of the pathways networks is present. Recent technological progress has raised the throughput and reliability of experimental techniques (typically based on yeast-2-hybrid assays) to a level potentially compatible with large-scale pathway reconstruction [9,7,6].

Pathway data is contained within databases, whose schemas reflect the kind of pathway category model they describe. Many databases already exist that represent and store various kinds of pathways and molecular interactions information. Some of the most established are KEGG, EcoCyc, BRENDA, WIT2 for Metabolic Pathways, CSNDB, AFCS, SPAD, BRITE and TransPath for Signaling Pathways, TRRD or TransPath for Gene Regulatory Networks, BIND, DIP, MIPS and GeneNet for protein interactions [1]. Some of these databases

Copyright © 2002 John Wiley & Sons, Ltd.

store information in flat file form, some are relational, while others are object-oriented or represent information using more complex data-structures or knowledge-representation techniques. Whatever the underlying storage and query engine type and its associated expressiveness, however, each database relies on a specific data model to structure its information.

A question asked by many scientists is whether it is at all possible to 'unite' all these models together into one schema. A unified database would in particular allow researchers to query multiple kinds of pathway information that may be associated, yet involve different types of pathways, for instance gene networks to regulate house-keeping metabolic enzymes. Such a view would greatly help in understanding what the downstream metabolic effects are, beginning from the gene regulation level.

One reason such direct unification appears difficult, however, is that the schemas for each category have been designed by separate scientific communities, to represent different biological phenomena with specific biases: metabolic pathways focus on step-by-step processing of biochemical products through catalysis, dividing the entities into mutable substrates and immutable enzymes. Classical representations of signal cascades [8] involve the serial activation (deactivation) of proteins that themselves may be activators (deactivators), so that they are simultaneously both operators and operands. Moreover, not only are many existing data models specific to a type of pathways, but most have been designed with specific applications in mind, such as pathway reconstruction, simulation, or annotation. Because of this unavoidable dependency between model and purpose, the idea of a unique 'one-size fits all' pathways model at the detailed 'relational schema' level may be unattainable.

At a higher level of abstraction, however, the most expressive 'common denominator' elements tend to emerge from practice; for instance, distinction between catalytic activities and enzymes is a necessity if one is to represent multiple catalytic influences on reactions. Ontologies are a way to describe in a formal and structured way such conceptual bricks and their interrelations; for example, the kinds of relationships between biochemical molecules such as allosteric modulation, competitive inhibition, covalent activation, non-covalent binding, catalysis, associative anchoring, complex stabilization, transcriptional binding, or receptor coupling. While the exact definition and scope of the word 'ontology' is still a subject of heated debate within the community, many researchers strongly feel the necessity of some kind of higher-level structured representation of pathway information [5], to allow for deeper analyses and functional inferences.

One of the foundational and ongoing activities of the BioPathways Consortium is to identify and categorize applications of computational representations of pathways information, assess the requirements these applications impose on data models, and assess the adequacy of existing formalisms – such as Petri Nets, process algebra (e.g., pi-calculus, ambient-calculus), and partial differential equations [4] – to the description of the various model classes. Another major area of activity of the Consortium is promoting the identification and specification of a set of commonly accepted ontologies that can work together with the above formalisms.

Standards for representing pathway information would indeed be beneficial to the development of pathway algorithms and tools. While the different application types that utilize pathway data in one form or another induce different requirements on the underlying models, many projects rely on the interplay between several applications, clearly underlining the need for a common data (and knowledge) interchange model. There will certainly be many more advanced applications developed in the near future, and the ease with which these can be realized will depend strongly on how much can be defined as a core set of guidelines and formalisms for pathways informatics.

Organizations exist that can help promote any such specifications and standards, including I3C (**http://www.i3c.org**), LSR-OMG (**http://www.omg.org/lsr**), and the Bio-Ontologies Consortium. Life science informaticists are also increasingly relying on the ease and expressiveness of XML-based exchange formats. Recently, the World-Wide-Web Consortium, which has defined and promoted XML standards, has also been working on the description of semantics for the content of XML documents called DAML (DARPA Agent Markup Language). This may offer a promising approach to organizing the complex data that can be associated with biological systems.

Within that context, the Biopathways Consortium aims at fostering the development of pathways informatics by a variety of means:

- synthesis and dissemination of scientific information on this emerging field, often from distant or

Copyright © 2002 John Wiley & Sons, Ltd.

unrelated sub-communities
- identification of scientific and IT challenges
- issuance of recommendations on standards
- support of industry-academia collaborations
- coordination with other life sciences groups

Several concurrent activities are currently being pursued by the Biopathways Consortium work-groups:

1. Formalisms: assessment of existing formalisms, data models, and exchange models, as well as recommendations on ontologies
2. Visualizations: identification and classification of tools and approaches
3. Database integration: the proposed PARIS project aiming at the design, development and maintenance of a public pathways information database
4. Pathway reconstruction: assessment and advancement of the algorithms for pathways information reconstruction from possibly heterogeneous sources of experimental data, as well as assessment of the nature, quality and quantity of the experimental data required.
5. Text mining from scientific literature: assessment, transfer and development of tools and techniques; an award received recently from Sun will allow the consortium to develop a test-bed for community research projects.

More information can be found on the BioPathways Consortium web site: **http://www.biopathways.org**

Even though this field is still nascent, there are pressing issues already that BioPathways can help address. A major driver is the need to interpret and organize newly produced genomic and proteomic data in the context of pathways and complex causal mechanisms. This data occurs in the form of micro-array data, mass spectrograms, metabolite profiles, and protein interaction networks. Model systems for analyzing this data and organizing it as supportive evidence for pathways and incorporation are urgently required. Specifically, gene expression research is increasingly being used to investigate the underlying network of mechanisms that regulate genes and may be the cause of many diseases. As new high-throughput technologies start producing new kinds of biological molecular data, these too must be consolidated into pathway models.

Pathways informatics is an interdisciplinary subject, involving rich modeling problems as well as difficult IT challenges. It is fueled mostly by the availability of information of such nature and quantity that exchange is an absolute necessity, and pulled by a variety of applications whose common points and key underlying concepts have not yet been clearly identified. Yet fruitful dialogue between biologists and computer scientists is an order of magnitude more important in dealing with pathways information than it was for sequence information, and will require the application of several of the above core concepts. Pathways informatics is a key enabler for the systems biology approach mentioned above. It is our hope that the Biopathways Consortium can be an enabler for pathways informatics.

### Acknowledgement

Our first thanks go to all active institutional or individual participants in BioPathways Consortium activities, a list unfortunately too long to reproduce here. We are especially indebted to Aviv Regev and Joanne Luciano, for many fruitful discussions on the field of pathways informatics, and for their help in organizing Consortium efforts.

Finally, the authors and Consortium co-founders wish to thank their institutions, Beyond Genomics and Hybrigenics, for their unfailing support, as well as Pierre Legrain and Donny Strosberg for a critical reading of the manuscript.

### References

1. Biopathways Consortium: **http://www.biopathways.org**
2. Bollobas B. 1998. *Modern Graph Theory*. Springer-Verlag: New York.
3. Ideker T, Thorsson V, Ranish JA, *et al.* 2001. Integrated genomic and proteomic analyses of a systematically perturbed metabolic network. *Science* **292**: 929–934.
4. Jamshidi N, Edwards JS, Church GM, Palsson BO. 2000. A computer model of human red blood cell metabolism. *Bioinformatics* **17**: 286–287.
5. Karp PD. 2001. Pathway databases: a case study in computational symbolic theories. *Science* **293**: 2040–2044.
6. Legrain P, Jestin JL, Schächter V. 2000. From the analysis of protein complexes to proteome-wide linkage maps. *Curr Opin Biotechnol* **11**: 402–407.
7. Rain JC, Selig L, De Reuse H, *et al.* 2001. The protein–protein interaction map of *Helicobacter pylori*. *Nature* **409**: 211–215.
8. Roberts CJ, Nelson B, Marton MJ, *et al.* 2000. Signaling and circuitry of multiple MAPK pathways revealed by a matrix of global gene expression profiles. *Science* **287**: 873–880.
9. Uetz P, Giot L, Cagney G, *et al.* 2000. A comprehensive analysis of protein-protein interactions in *Saccharomyces cerevisiae*. *Nature* **403**: 623–627.

Copyright © 2002 John Wiley & Sons, Ltd. 
